# Pathological Impairment, Cell Cycle Arrest and Apoptosis of Thymus and Bursa of Fabricius Induced by Aflatoxin-Contaminated Corn in Broilers

**DOI:** 10.3390/ijerph14010077

**Published:** 2017-01-13

**Authors:** Xi Peng, Shiping Bai, Xuemei Ding, Keying Zhang

**Affiliations:** 1 Institute of Animal Nutrition, Key Laboratory for Animal Disease-Resistance Nutrition of China Ministry of Education, Sichuan Agricultural University, Ya’an 625014, Sichuan, China; pengxi197313@163.com (X.P.); shipingbai@aliyun.com (S.B.); dingxuemei0306@163.com (X.D.); 2 College of Life Science, China West Normal University, Nanchong 637009, Sichuan, China

**Keywords:** aflatoxin, comparative lesion, thymus, bursa of Fabricius

## Abstract

This study aimed to evaluate the comparative effects of aflatoxin-contaminated corn on the thymus and bursa of Fabricius (BF) in chickens by detecting histopathological lesions, cell cycle phase distribution and apoptosis. A total of 900 COBB500 male broilers were randomly allocated into five groups. The experiment lasted for six weeks and the five dietary treatments consisted of uncontaminated corn (control), 25% contaminated corn, 50% contaminated corn, 75% contaminated corn and 100% contaminated corn groups. The gross changes showed the decreased size of the thymus and BF, as well as the pale color of the BF in the broilers after aflatoxin contaminated diet exposure. There were more nuclear debris in the thymus and BF of birds in the 50%, 75%, and 100% contaminated corn groups, but the pathological impairments of the BF were more obvious than those of the thymus, which showed as more obvious lymphocyte depletion and the proliferation of reticulocytes and fibroblasts. At 21 days of age, the percentage of thymocytes and BF cells in the G2M phase was increased in a dose-dependent manner in the four AFB-contaminated corn groups. However, at 42 days of age, dietary AFB_1_ induced cell cycle perturbation at the G0G1 phase in thymocytes, but at the G2M phase in BF cells. The increased percentage of apoptotic cells in the thymus and BF were similarly observed in the AFB groups. According to these results, the severity of histopathological lesions may be correlated with the different sensitivity of the two central immune organs when exposed to AFB; different arrested cell cycle phases suggest that different mechanisms may be involved in the lesions of the thymus and BF, which need to be further researched.

## 1. Introduction

Mycotoxins are secondary fungal metabolites that can be produced in maize and other food commodities [1]. They can cause adverse health effects in humans and animals because of their toxicological effects [2]. Aflatoxins are a group of cytotoxic and carcinogenic mycotoxins produced by *Aspergillus flavus*, fungi that commonly contaminate feed ingredients during storage. The most important member of the group is aflatoxin B_1_ (AFB_1_), which has a range of biological toxicities, including acute hepatic toxicity, teratogenicity, mutagenicity and carcinogenicity [3]. It caused hepatotoxicity in all species examined thus far [4]. The carcinogenic potency of AFB_1_ has been observed in many species of animals, including nonhuman primates, rodents, birds and fish [5,6,7,8]. AFB toxicity can result from various mechanisms, such as oxidative stress, apoptosis or cell cycle blockage [9,10,11]. It is known that immunosuppression can result in a greater rate of tumor progression [12], and previous studies have reported that AFB_1_ can induce immune toxicity in various animal species [13,14,15,16,17,18]. In vitro and in vivo studies have shown that AFB_1_ exerted immunotoxic action on cell-mediated immunity by reducing the number of circulating lymphocytes [19,20], inhibiting the proliferation of lymphocytes [21,22], and altering the activity of natural killer (NK) cells and the expression of cytokines [23].

To our knowledge, there are some reports [19,24,25,26] of associated impaired immune systems with the histological structure of the immune organ exposed to aflatoxin, but most of these were focused on single mycotoxin (AFB_1_). In our previous study, ultrastructural changes of the thymus and bursa of Fabricius (BF) were also observed in chicks fed a diet including corn mainly contaminated with AFB_1_ and AFB_2_ [27]. In avian species, the thymus and BF are responsible for the establishment and maintenance of T cell and B cell compartments, respectively. So domestic fowl, for example chickens or ducks, maybe used as a good model for studying of the effects of toxins on T lymphocytes or B lymphocytes in vivo. In this study, through determining the histological lesions, cell cycle phase distribution and apoptotic percentages of the thymus and BF, our objective was to evaluate the tissue basis and correlated mechanisms of immunosuppression induced by naturally AFB-contaminated corn.

## 2. Materials and Methods

### 2.1. Animals, Diet, and Study Design

Nine hundred one-day-old COBB500 chickens were randomly assigned to five experimental groups with six replicates per groups and 30 birds per replicates. The birds were housed in the cages for 42 days at the Animal Nutrition Institute of Sichuan Agricultural University in China. Chicks were freely access to food and water. Room lights were set on a 24 h continuous schedule, temperature was initially maintained at 31 °C and gradually lowered by 2 °C each week until 21 °C, and relative humidity were maintained between 65% and 67%. The animal experiment was conducted in accordance with guidelines approved by Animal Health and Care Committee of Sichuan Agricultural University (Code: 2012-024).

The control animals were fed on the corn-soybean basal diet. Nutritional requirements of the diet were adequate according to National Research Council (1994) and Agricultural Trade Standardization of China (NY/T33-2004). The composition and nutrient levels of the diet were described previously [28]. The basal control diet was not contaminated with AFB_1_ and AFB_2_. The four treated groups were given diets in which the ratio of naturally contaminated corn was 25%, 50%, 75%, and 100%, respectively. By the method of high performance liquid chromatography (Agilent 1100, Agilent Technologies, Santa Clara, CA, USA), the contents of mycotoxins, including aflatoxin B_1_ (AFB_1_), aflatoxin B_2_ (AFB_2_), aflatoxin G_1_ (AFG_1_), aflatoxin G2 (AFG_2_), T-2 toxin, Deoxynivalenol (DON), Zearalenone (ZEN), Ochratoxin A (OTA), and Fumonisin B_1_ (FB_1_) was detected as described previously [27]. The detection limits of above mycotoxins were 2 µg/kg for AFB1, 0.8 µg/kg for AFB_2_, 2.5 µg/kg for AFG_1_, 1.5 µg/kg for AFG_2_, 100 µg/kg for T-2 toxin, 300 µg/kg for DON, 100 µg/kg for ZEN, 30 µg/kg for OTA, and 200 µg/kg for FB_1_ [29].

The results showed that naturally contaminated corn used in the diet was mainly contaminated with AFB_1_ and AFB_2_. The AFB_1_ contents in diets were 16.3–82.4 µg/kg in the starter period and 34.3–134 µg/kg in the grower period (Table 1). The AFB_2_ concentrations in diets were 3.15–14.2 and 6.17–23.6 µg/kg in the starter and grower periods, respectively (Table 1). The contents of other mycotoxins (including AFG_1_, AFG_2_, DON, ZEA, OTA, T-2 toxin, and FB_1_) were below the limit of detection. These diets and water were provided ad libitum throughout the 42 days of experimentation.

### 2.2. Pathological Observation

At 14, 21, 28, and 42 days of age during the experiment, six birds in each group were euthanized and necropsied. And then, thymus and BF were dissected form each chick, and fixed in 4% buffered formaldehyde and routinely processed in paraffin. Thin sections (5 μm) of each tissue were sliced from each block and mounted on glass. Slides were stained with hematoxylin and eosin Y. Histological slides were examined on an Olympus light microscope.

### 2.3. Cell Cycle Phase Detection by Flow Cytometry

At 21 and 42 days of age during the experiment, six birds in each group were euthanized. Thymus and BF were dissected from each chick and immediately minced with surgical scissors. The cell suspension was filtered through a 300-mesh nylon mesh, washed twice with 0.1 M (pH 7.4) cold phosphate buffered saline (PBS). And then, the cells were resuspended in PBS at a concentration of 1 × 10^6^ cells/mL. The 1 mL suspension was transferred to a 5 mL culture tube and centrifuged at 200× *g* for 5 min. The supernatant was discarded, and 1 mL PI staining solution (5 μL/mL Propidium iodide, 0.5% Triton X-100, 0.5% RNase, PBS) was added. The cells were gently vortexed and incubated for 20 min at room temperature (25 °C) in the dark. Then 2 mL PBS were added and centrifugal elutriation performed once. The supernatant was discarded. The cells were re-suspended in 0.5 mL PBS and the cell cycle phases were analyzed by flow cytometry (FACSCalibur, BD Company, Franklin Lakes, NJ, USA).

### 2.4. Annexin V Apoptosis Detection by Flow Cytometry

The above mentioned cells were resuspended in 1× binding buffer (Cat. No. 51-66121E) at a concentration of 1 × 10^6^ cells/mL. One hundred μL of the solution was transferred to a 5-mL culture tube, and then 5 μL of Annexin V-FITC (Cat. No. 51-65874X) and 5 μL of PI (Cat. No. 51-66211E) were added. The cells were gently vortexed and incubated for 15 min at room temperature (25 °C) in the dark. 400 μL of 1× binding buffer were added to each tube and analyzed by flow cytometry within 1 h.

## 3. Results

### 3.1. Histopathological Lesions

**Figure 1:**
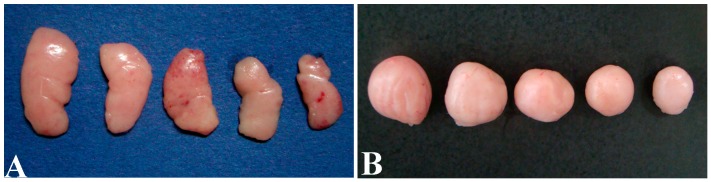
Gross changes in the size of the thymus (**A**) (first lobus on the right) and BF (**B**) of the chickens at 21 days of age. From left to right: organs in the control group, 25%, 50%, 75% and 100% groups.

Macroscopically, thymuses were lightly reduced in size in the 25%, 50%, 75% and 100% groups (Figure 1A). The BF in the AFB-contaminated groups showed obvious atrophy with decreased volume and a pale color. Most typical changes were observed at 21 days of age, which showed that the volume of the BF was decreased with the increased dietary AFB level (Figure 1B). The lesions of the thymus and BF in AFB-contaminated groups were all alleviated at 42 days of age.

Histomorphological changes of hematoxylin- and eosin-stained thymus and BF sections following AFB exposure were evaluated. Representative photomicrographs of the three immune organs were chosen to illustrate key observation here.

**Figure 2:**
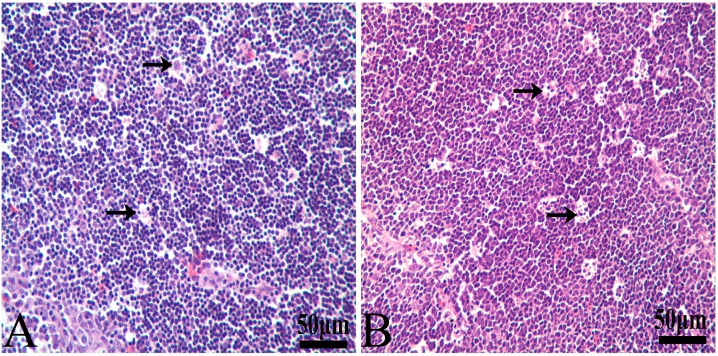
Photomicrographs of hematoxylin- and eosin-stained chicken thymus section at 21 days of age. (**A**) Control group. A little nuclear debris (→) can be seen around individual reticular epithelial cells; (**B**) 100% group. Compared with (**A**), there is more nuclear debris (→) around reticulocytes. Bars = 50 µm.

In the control group, reticular epithelial cells are scattered in the medulla, and a little nuclear debris can be seen around individual reticular epithelial cells (Figure 2A). In the four AFB-contaminated groups, the amount of nuclear debris had an increasing tendency with the increased content of the mycotoxin. Most obvious lesions were observed at 14 and 21 days of age. In the 75% and 100% groups, there was much nuclear debris around the reticulocytes (Figure 2B).

**Figure 3:**
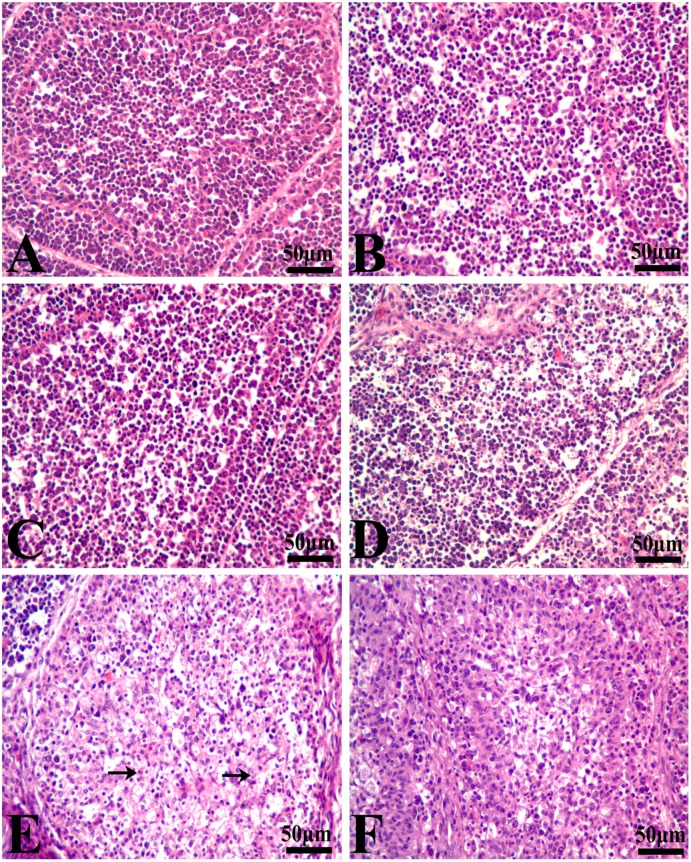
Photomicrographs of hematoxylin- and eosin-stained chicken BF section. At 14 days of age, when compared with that of the control group (**A**), nuclear debris was gradually increased in the BF of chickens in the 50%, 75% and 100% groups (**B**–**D**, respectively). At 21 days of age, histologic lesions became deteriorated. In the 100% group, more nuclear debris (→) was observed (**E**). At 42 days of age, the BF in the 100% groups still showed decreased lymphocytes and increased reticulocytes and fibroblasts (**F**). Bars = 50 µm.

There were no obvious lesions in the BF of the 25% group when compared with those of the control group (Figure 3A) during the 42-day experiment. At 14 days of age, necrotic lymphocytes in the cortex and medulla of the lymphoid follicles in the BF were increased in number in the 50%, 75%, and 100% groups (Figure 3B–D). The most obvious lesions were observed in the 100% group. It was difficult to see the epithelium separating the medulla and cortex in the follicles owing to the massive nuclear debris (Figure 3E). At 21 days of age, the histologic lesions became deteriorated. More nuclear debris was observed in the 50%, 75%, and 100% groups, and hypocellularity was especially obvious in the 100% groups. It is interesting that histological lesions of the BF in the mycotoxin-contaminated groups were all obviously alleviated at 42 days of age. At 42 days of age, no lesions were observed in the 50% and 75% groups, but the BF in the 100% group still showed atrophy with a thinner cortex, a wider medulla and increased reticulocytes and fibroblasts (Figure 3F).

### 3.2. Cell Cycle Phase Distribution of Immune Organs

**Figure 4:**
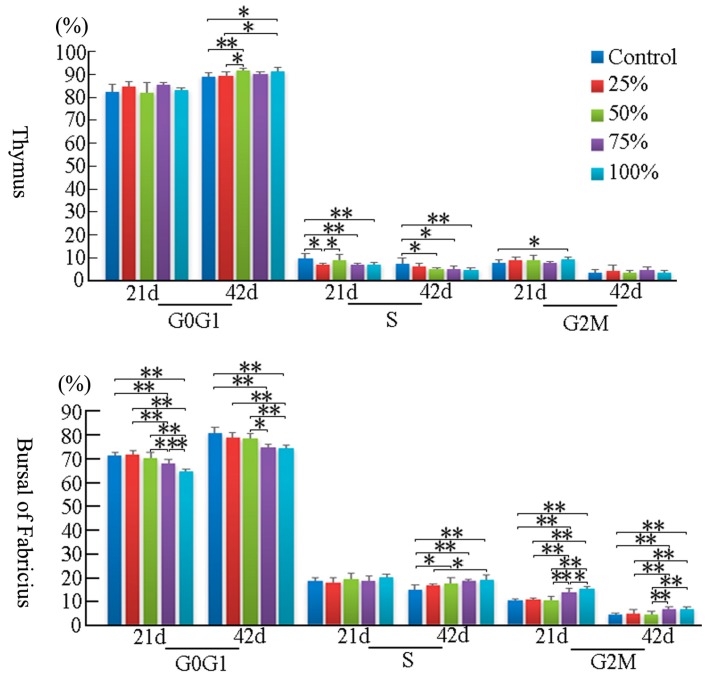
Effects of AFB-contaminated corn on cell cycle phase distribution of thymocytes and BF cells in chickens. ** represents the significant difference (*p* < 0.01). * represents difference (*p* < 0.05).

**Figure 5:**
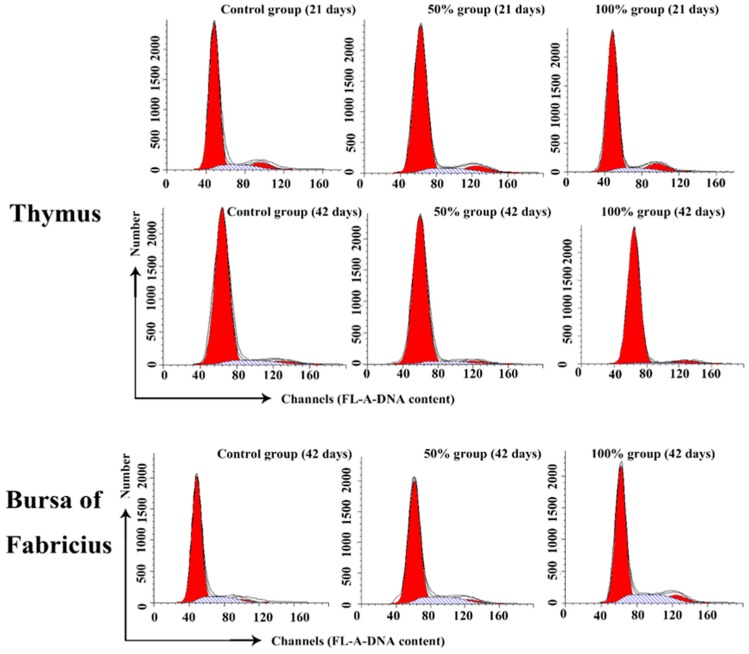
Histogram of cell cycle by FCM. In the thymus, the increase of G2M phase cells at 21 days of age and the decrease of S and G2M phase cells in AFB-contaminated groups at 42 days of age are obviously seen. In the BF, the increase of S and G2M phase cells can be observed.

At 21 days of age, the percentage of G2M thymocytes in the 100% group was higher than that in the control group (*p* < 0.05), and the percentage of S phase thymocytes was decreased in a dose-dependent manner. However, at 42 days of age, the percentage of thymocytes in the G2M phase had no obvious changes among the five groups. When compared to the control group, the thymocytes in the G0G1 phase were significantly increased in the 50% and 100% groups (*p* < 0.01), and the cells in S phase were markedly decreased with a dosage-dependent trend (*p* < 0.01 or *p* < 0.05). The results are shown in Figure 4 and Figure 5.

At 21 days of age, the percentages of BF cells in the G0G1 phase were decreased significantly in the 75% and 100% groups (*p* < 0.01), and the cells in the G2M phase were increased obviously in the corresponding groups when compared to the control group (*p* < 0.01), while there were no obvious changes on the percentage of the S phase BF cells between different experimental groups. Furthermore, changes of BF cells were found to have the same increased trend in the G2M and S phases at 42 days of age. However, the percentages of bursal cells in the G0G1 phase were significantly decreased as the dietary aflatoxin level increased at 42 days of age when compared to the control group (*p* < 0.05 or *p* < 0.01). The results are shown in Figure 4 and Figure 5.

### 3.3. Annexin V-FITC Staining Assay by Flow Cytometry

**Figure 6:**
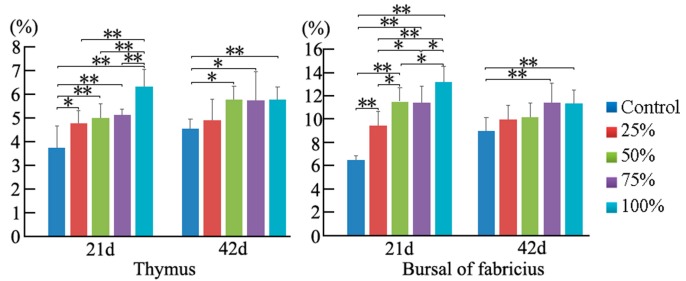
Effects of AFB-contaminated corn on the percentages of apoptotic cells in the thymus and BF of chickens. ** represents the significant difference (*p* < 0.01). * represents difference (*p* < 0.05).

**Figure 7:**
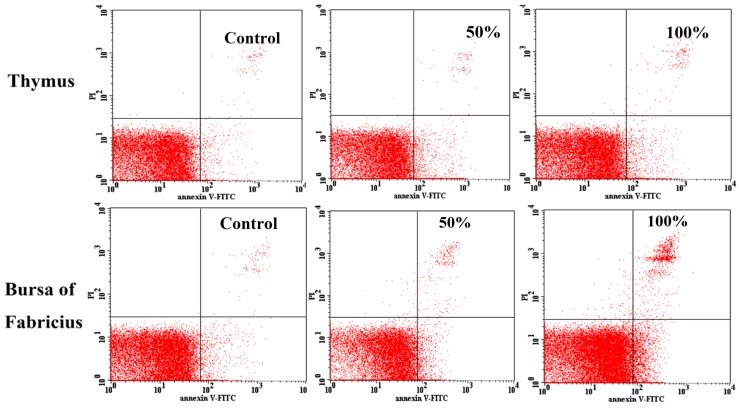
Cells stained positive for Annexin-V-FITC are undergoing apoptosis. Quadrantal diagram shows that increased apoptotic thymocytes and BF cells in the 50% and 100% groups are undergoing apoptosis when compared with those in the control group.

As shown in Figure 6, the percentages of apoptotic thymocytes and BF cells were increased as dietary aflatoxin level increased. At 21 days of age, when compared with those in the control group, the percentages of apoptotic thymocytes in the 25%, 50%, 75%, and 100% groups were increased (*p* < 0.01 or *p* < 0.05), and the percentages of apoptotic BF cells in 50%, 75% and 100% groups were significantly increased (*p* < 0.01). At 42 days of age, the percentages of apoptotic thymocytes in the 50%, 75%, and 100% groups, and apoptotic BF cells in the 75% and 100% groups were higher (*p* < 0.01 or *p* < 0.05) than those in the control group. The quadrantal diagram analyzed by the flow cytometer showed that more thymocytes and BF cells in these AFB-contaminated groups were undergoing apoptosis (Figure 7).

## 4. Discussion

The thymus and BF of avian species are known as the central immune organs for the diversification and maintenance of T cells and B cells, respectively, so this study aimed to study the comparative toxic effects of dietary aflatoxin on T and B lymphocytes in vivo by using a chicken model. The gross changes showed the decreased size of the thymus and BF, as well as the pale color of the BF in the broilers after AFB-contaminated diet exposure, which was in agreement with other research [24,25,26]. Histopathologically, there was more nuclear debris in the thymus and BF of birds in the 50%, 75%, and 100% groups, which indicated that excessive necrotic or apoptotic cell death of T and B lymphocytes occurred in a dose-dependent manner in the thymus and BF of broilers, respectively. The histological damage of the thymus and BF suggested the impairment of cellular and humoral immune function, respectively. Comparatively, the pathological impairments of the BF were more obvious than those of the thymus in chickens exposed to AFB-contaminated diets, which appeared as more nuclear debris, obvious lymphocyte depletion and the proliferation of reticulocytes and fibroblasts, suggesting that the BF might be more sensitive to aflatoxins than the thymus. However, the histological lesions of the thymus and BF were all alleviated at 42 days of age, suggesting increased tolerance with the growth of the chickens. According to our research on ultrastructural changes [27] and the results of apoptosis in this study, the histological lesions were closely related to the upregulated apoptosis of cells in the thymus and BF.

The percentage of cells in different cell division phases can be analyzed by flow cytometry [30]. Previous studies have shown that aflatoxins can induce cell cycle arrest in different phases, depending on the cell type and growth conditions [9,31,32,33,34]. In our present study, the percentage of thymocytes and BF cells in the G2M phase was increased in a dose-dependent manner at 21 days of age. However, at 42 days of age, exposure to dietary AFB_1_ induced cell cycle perturbation in the G0G1 phase in thymocytes, but in the G2M phase in BF cells. Scott et al. reported that AFB_1_-treated thymocytes were accumulated in the G2M phase in vitro [33], and our previous research showed that G2M and G0G1 phase blockage could be observed in the spleen of chickens exposed to AFB-contaminated corn [34]. However, some other researchers showed that aflatoxins can induce cell cycle arrest of the S phase in renal cells and macrophages [9,31]. These studies and our study suggest that the accumulated cell phases are different in AFB-induced cell cycle blockage, which might be related to different cells or different in vivo and vitro conditions. The results of our present study showed an interesting finding: that dietary AFB initially caused an accumulation of the G2M phase, while there was a later accumulation of the G0G1 phase in thymocytes, but the BF cells were consistently arrested in the G2M phase at 21 and 42 days of age, suggesting that the characteristics of cell cycle arrest could be changeable in the same kinds of cells with the increase of the AFB exposure time.

Apoptosis, or programmed cell death, is a major control mechanism by which cells die if cell injuries are not repaired [35,36]. DNA damage and externalization of phosphatidylserine from the inner to the outer leaflet of the plasma membrane are the most important biomarkers of apoptotic cells. With the flow cytometry method, the early apoptotic cells can be analyzed through identifying exposed phosphadylserine on the cell surface [37]. In the present study, the percentage of apoptotic cells in the thymus and BF in the AFB groups was higher than that in the control group. Our results indicated that excessive apoptosis of thymocytes and BF cells may result in decreased immune function, which could explain the tissue basis of AFB-induced immunosuppression to some degree. Based on our recent study [38], AFB_1_ increased the percentage of apoptotic cells and increased the expression of Caspase-3 in both the thymus and BF in chickens. In our recent research with qPCR detection, although mitochondrial pathway–related genes were involved both in the apoptosis of thymocytes and bursal cells, the death receptor pathway and endoplasmic reticulum pathway were triggered in the apoptotic procedure of thymocytes and bursal cells, respectively [15,16]. Accordingly, the expanded endoplasmic reticulum with a high electron density (accumulated immune globulin) was only observed in the lymphocytes of the BF. When chicks were fed corn mainly contaminated with AFB_1_ and AFB_2_, vacuolated mitochondria with degenerated cristae were observed in the lymphocytes of the thymocytes and BF cells, which was in line with previous research [15,16,34]; however, greater numbers of secondary lysosomes in BF cells were observed, which was different from the condition resulting from only AFB1 intake [16]. These results suggested that different kinds of mechanisms might be related to different immune cells and mycotoxin components, which must to be clarified further.

## 5. Conclusions

According to the results of this study, together with the above discussion, the severity of histopathological lesions suggests that the BF might be more sensitive to aflatoxins than the thymus, and different accumulated cell phases in the thymocytes and BF cells show that different mechanisms maybe involved in the lesions of the thymus and BF, which need to be researched further.

## Figures and Tables

**Table 1:** Mycotoxin concentrations in diet and corn (air-dry basis μg/kg).

Diet ^1^	Control	25%	50%	75%	100%	Control Corn	Contaminated Corn
1–21 days							
AFB_1_	ND ^2^	16.3	36.9	45.6	82.4	ND	149.6
AFB_2_	ND	3.15	6.38	7.86	14.2	ND	24.2
22–42 days							
AFB_1_	ND	34.3	69.3	95.2	134	ND	229
AFB_2_	ND	6.17	12.1	17	23.6	ND	37.8

^1^ Control = diet with control corn; 25% = diet with 25% naturally contaminated corn; 50% = diet with 50% naturally contaminated corn; 75% = diet with 75% naturally contaminated corn; 100% = diet with 100% naturally contaminated corn; ^2^ ND = not detectable.
